# Assessing Creatine Supplementation for Neuroprotection against Perinatal Hypoxic-Ischaemic Encephalopathy: A Systematic Review of Perinatal and Adult Pre-Clinical Studies

**DOI:** 10.3390/cells10112902

**Published:** 2021-10-27

**Authors:** Nhi Thao Tran, Sharmony B. Kelly, Rod J. Snow, David W. Walker, Stacey J. Ellery, Robert Galinsky

**Affiliations:** 1 School of Health & Biomedical Sciences, STEM College, RMIT University, Melbourne 3083, Australia; nhi.tran@hudson.org.au (N.T.T.); david.walker@rmit.edu.au (D.W.W.); 2 The Ritchie Centre, Hudson Institute of Medical Research, Melbourne 3168, Australia; Sharmony.Kelly@monash.edu (S.B.K.); stacey.ellery@hudson.org.au (S.J.E.); 3 Department of Obstetrics & Gynecology, Monash University, Melbourne 3168, Australia; 4 Institute for Physical Activity & Nutrition, Deakin University, Melbourne 3125, Australia; rod.snow@deakin.edu.au

**Keywords:** neuroprotection, hypoxic ischaemic encephalopathy, brain injury, perinatal encephalopathy, creatine, phosphocreatine

## Abstract

There is an important unmet need to develop interventions that improve outcomes of hypoxic-ischaemic encephalopathy (HIE). Creatine has emerged as a promising neuroprotective agent. Our objective was to systematically evaluate the preclinical animal studies that used creatine for perinatal neuroprotection, and to identify knowledge gaps that need to be addressed before creatine can be considered for pragmatic clinical trials for HIE. Methods: We reviewed preclinical studies up to 20 September 2021 using PubMed, EMBASE and OVID MEDLINE databases. The SYRCLE risk of bias assessment tool was utilized. Results: Seventeen studies were identified. Dietary creatine was the most common administration route. Cerebral creatine loading was age-dependent with near term/term-equivalent studies reporting higher increases in creatine/phosphocreatine compared to adolescent-adult equivalent studies. Most studies did not control for sex, study long-term histological and functional outcomes, or test creatine post-HI. None of the perinatal studies that suggested benefit directly controlled core body temperature (a known confounder) and many did not clearly state controlling for potential study bias. Conclusion: Creatine is a promising neuroprotective intervention for HIE. However, this systematic review reveals key knowledge gaps and improvements to preclinical studies that must be addressed before creatine can be trailed for neuroprotection of the human fetus/neonate.

## 1. Introduction

Hypoxic ischaemic encephalopathy (HIE) remains a major cause of neonatal mortality and morbidity with lifelong disabilities. The overall incidence of HIE at term is reported to be 25/1000 live births annually, however the majority of these cases were categorized as mild HIE [1]. Moderate to severe HIE is seen in approximately 4/1000 live births annually [2]. Although HIE occurs in a minority of births, when it is present, irrespective of severity, it is a substantial contributor to lifelong disability [3,4]. Although mild therapeutic hypothermia (cooling) for HIE in near term and term infants is now well established to improve survival without disability in developed countries, current hypothermia protocols are only partially protective, such that approximately 30% to nearly half of infants still die or survive with disability despite cooling [5,6]. In developing countries, where the incidence and burden of HIE related morbidity and mortality is highest [7,8], there is no effective treatment for HIE, and preliminary evidence suggests that therapeutic hypothermia may not be effective [9,10,11]. Thus, there is an urgent unmet need for additional treatments to further improve outcomes.

Creatine has emerged as a promising neuroprotective agent in the adult and developing brain. Creatine is an amino acid derivative involved in cellular energy production [12], but other beneficial properties of creatine have been identified, including antioxidative, lipid membrane stabilisation, and antiexcitotoxicity through interaction with glutamate and GABA_A_ receptors [13]. In healthy adult females, creatine supplementation has a good safety profile [14] and a prospective cohort study of maternal creatine supplementation and postnatal outcomes is ongoing (ACTRN12618001558213) [15]. Thus, if maternal dietary creatine supplementation proves to be safe for the mother and neonate, antenatal and/or postnatal creatine supplementation for neuroprotection of the foetus and/or newborn could provide an inexpensive and easily used intervention for HIE that can be applied in high- and low-resource settings in developed and developing countries.

The purpose of this review is to systematically evaluate the preclinical in vivo studies on creatine for perinatal neuroprotection and identify knowledge gaps that need to be addressed before creatine supplementation could be considered practically for clinical trials for HIE.

## 2. Materials and Methods

### 2.1. Literature Search

This systematic review was conducted according to the Preferred Reporting Items for Systematic Reviews and Meta-Analyse (PRISMA) guidelines [16] (Supplementary Tables S1 and S2). Studies were searched for on PubMed, EMBASE and OVID MEDLINE on 20 July 2021 and 20 September 2021 with no restrictions on date of publication. The search strategy comprised the following Medical Subject Headings (MeSH) or keywords specific to each database: (1) Hypoxia-ischaemia, brain; Hypoxia, brain; Ischaemia, brain; Stroke; (2) Creatine; (3) Brain/Brain injury; (4) Animal. Literature search terms are outlined in Table 1. Reviews, conference abstracts, articles written in a language other than English or for which no translation was available were excluded. Search results were collated, and duplicate articles manually removed. Abstracts were initially screened by an unbiased investigator from our study group (NTT) and duplicated by another investigator (SBK). Full-text articles were then retrieved and assessed for final qualitative synthesis. The inclusion of adult studies, in addition to perinatal studies, in this systematic review is justified by the following: 1. The mechanisms underpinning adult and perinatal HIE are broadly similar and 2. Preclinical adult studies of HIE have produced many of the parameters (e.g., dosages and treatment regimens) that informed the design of the perinatal studies. Thus, we believed including adult studies would help further inform where the existing gaps in knowledge lie in relation to the therapeutic potential of creatine for perinatal HIE.

### 2.2. Selection Criteria

Articles were included if they met the following criteria: (1) used a hypoxic and/or ischemic insult to induce brain injury in vivo; (2) creatine or phosphocreatine, creatine monohydrate, disodium phosphocreatine, and anhydrous creatine were administered as a treatment regardless of dosage, timing, regime, or administrative route; (3) reported the experimental outcomes of creatine intervention on brain pathology and/or behaviour and/or neurophysiology; and (4) included a comparison to a vehicle control group. Studies were excluded if: (1) an adjunct therapy was administered where the effect of creatine supplementation was not independently assessed; (2) a creatine analogue, synthesized through modification of the creatine salt with other compounds, was administered; and (3) the injury model did not involve hypoxia and/or ischaemia.

### 2.3. Data Extraction

Extracted data included the general study design (animal species, sample size, sex), age at birth of preclinical model (preterm vs. term equivalent), HIE model, creatine intervention (regime, timing, dosing), and experimental outcomes. Studies that tested the effect of multiple interventions, i.e., pre vs. post HI treatment, were regarded as separate studies.

For assessment of neuroprotection, the experimental outcomes measures included (1) functional neurologic outcomes, reported through behavioural, cognitive, sensorimotor or mobility tests, or electrophysiological tests; (2) histological examination of processed brain tissue; and/or (3) brain metabolism related to bioenergetic effects. Given the wide range of primary outcomes assessed, the differences in timing and dosage of creatine treatment, and the different preclinical modelling, which included differences in age and injury protocol, a meta-analysis was not possible and therefore a narrative summary of results is presented.

### 2.4. Risk of Bias

A risk of bias assessment for final selected studies was conducted using the SYRCLE Risk of Bias (RoB) tool [17]. The SYRCLE’s RoB tool assesses the quality of animal studies (e.g., randomization and blinding procedures in study design) with the aim of critically appraising preclinical research for translation into clinical practice. The 10 RoB assessment domains were scored as either “yes” for low risk of bias, “no” for high risk of bias, or “unclear” if the experimental methods did not explicitly address the domain assessment.

## 3. Results

### 3.1. Search Results

**Figure 1:**
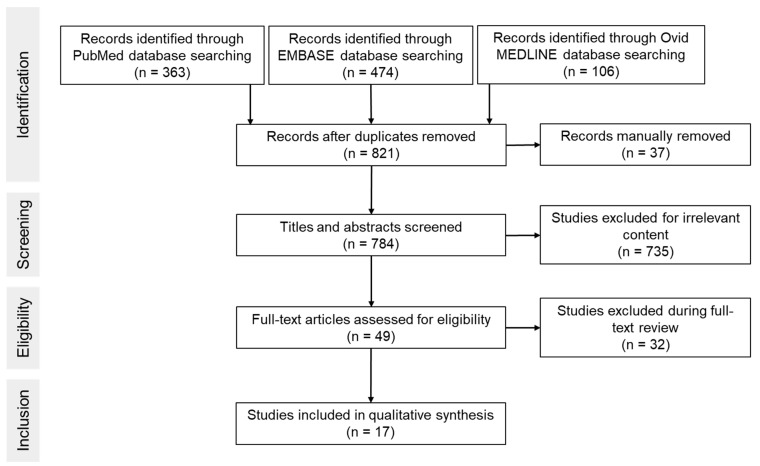
Flowchart of search process.

Figure 1 shows an overview of the screening process. Searches through databases PubMed, EMBASE and OVID MEDLINE resulted in 363, 474 and 106 articles, respectively (Table 1). A total of 784 studies were screened by title and abstract. Seven hundred and sixty-seven studies were excluded due to one or more of the following: no creatine treatment, creatine analogues used, absence of HI induced injury, ex vivo studies or histological and/or behavioral outcomes were not assessed. Thus, a total of 17 publications were included in the analysis.

### 3.2. Description of Included Studies

Eight out of 17 studies were performed in perinatal models of HIE (Table 2). Six out of 8 studies were conducted in rodents between postnatal days (P)7 and P20 [18,19,20,21,22,23,24], which is considered comparable to human brain development between near-term/term and late infancy [25,26]. One study was conducted in term spiny mice which in terms of brain development are broadly comparable to the term human [27]. One study was conducted in newborn rabbits (P5–P30) [21], which are broadly comparable to human brain development between term and late infancy [28]. Most of the rodent studies (5/8) used the Rice-Vanucci model of unilateral carotid artery ligation followed by a period of moderate hypoxia [18,19,22,23,24]. This paradigm of perinatal brain injury is associated with infarction of the middle cerebral artery region ipsilateral to the ligated carotid artery, with no overt cell death in the contralateral cerebral hemisphere [25]. Most of these studies (4/5) examined infarct area to assess neuronal loss/survival/severity of the insult, 1/5 used MRI to assess tissue edema [18]. In spiny mice, global hypoxia was induced by isolating the pregnant uterus in a warm saline bath for 7.5 min which resulted in widespread lipid peroxidation and a marked increase in apoptosis within cortical and thalamic regions [27]. One study in newborn rabbits and one study in newborn rats used prolonged moderate hypoxia to examine the impact of creatine on hypoxia-induced seizures [20,21] (Table 2).

Nine out of 17 studies were performed in adult models of HI (Table 3). One study included both prophylaxis and rescue treatment protocols and, in turn, was subdivided into separate studies [29], resulting in a total of 10 individual adult studies. Thus, for the purpose of reporting on the treatment regime, timing and outcomes we summarize the adult data based on the individual adult studies (*n* = 10). As the subdivided studies used the same species, developmental age, type and duration of temperature monitoring and reporting of sex; these parameters are summarized based on the number of original adult studies (*n* = 9).

All of the adult studies (9/9) were conducted in rodent models of cerebral ischaemia using either (1) global ischaemia induced by bilateral carotid artery occlusion or (2) focal ischaemia using middle cerebral artery occlusion (Table 3).

### 3.3. Regime and Timing of Creatine Delivery

Five out of 8 perinatal studies administered creatine before HI (pretreatment) [18,20,21,27]. One out of 5 pretreatment studies administered creatine via the maternal diet for 18 days before inducing intrapartum hypoxia and was associated with improved survival rates (from 45 to 59%) after birth asphyxia, with the increase in survival greater for males, and with improved histological outcomes for both males and females [27]. The remaining 4 studies in either P7 rats, P10–20 rats, or rabbits (P5–P30) administered creatine to the neonate via daily subcutaneous injections (3g/kg) for 3 days before HI [18,20]. Two studies reported improved survival after hypoxia and/or functional outcomes with treatment after hypoxia [20,21] and 1 study reported reduced brain oedema [18]. One out of 8 studies administered creatine via subcutaneous injections (3g/kg) before (at −64 h, −40 h, −16 h) and after (+3 h) HI and reported reduced histological injury with treatment [19]. Three out of 8 studies administered creatine to newborn mice via dietary/oral supplementation (1–3%) starting at 10 days after HI [22,23,24], 1 study reported no improvements in histological or functional outcomes [22], 1 reported improved function but no improvements in histological outcomes [23], and 1 reported improved histological and functional outcomes [24].

Of the 9 adult studies identified, 1 study used multiple interventions i.e., pre vs. post HI treatment and therefore was separated into 2 studies. This yielded a total of 10 individual adult studies. Seven out of 10 individual adult studies, all in rodents, administered creatine before HI. Of these, 4 used dietary supplementation (0.5–2%) for between 4 weeks and 10 days before HI [30,32,33,34], 2 studies used IV infusion between 60 and 30 min before HI [36,37], and 1 used intra-peritoneal creatine but the timing of administration before HI was not specified [31]. Six out of 7 studies reported that creatine was associated with neuroprotection. Two out of 10 studies administered creatine before and after HI, both reported neuroprotective effects [29,35]. One study used creatine immediately after (within 30 min) HI and reported improved histological outcome but no improvement in neurological function as assessed using a battery of tests for muscle strength and sensorimotor control [29].

### 3.4. Measurement of Creatine in the Brain

Four out of eight perinatal studies measured creatine and/or phosphocreatine (PCr) levels within the brain following creatine supplementation using either magnetic resonance spectroscopy or high-performance liquid chromatography [18,19,20,21]. In newborn rats, daily subcutaneous injections of creatine for 3 days before hypoxia was associated with increased cerebral PCr/nucleoside triphosphate (NTP) at P10 (by approximately 40%) but not at P20 [20]. Furthermore, Berger et al. reported an increase in creatine (by approximately 50%) and phosphocreatine (by approximately 45%) in the cerebral cortex of P7 rats at 6 h after a single subcutaneous creatine injection [19]. In rabbit pups aged between P5 and P30, daily subcutaneous injections of creatine for 3 days before hypoxia increased brain PCr/NTP ratios between P5 and P20 compared to controls (by approximately 60–30%). With increasing postnatal age, there was a reduction in the capacity for brain PCr/NTP to be increased in creatine-treated pups compared to controls [21].

Four out of nine adult studies measured cerebral creatine levels. Prass et al. showed that 3 weeks of dietary (0.5–2%) creatine supplementation did not increase cerebral creatine and phosphocreatine levels in 7-week-old mice [30]. In contrast, two studies reported a 13–20% increase in total brain creatine:choline after 10 days of dietary (2%) creatine supplementation compared to non-supplemented rodents. However, the age at which creatine was administered was not reported [33,34]. One study reported that cerebral creatine levels were increased by 17% in the ipsilateral (ischaemic) but not the contralateral (non-ischaemic) cerebral hemisphere 30 min after the ischaemic episode in mice that were fed a 2% creatine-supplemented diet for 4 weeks [32].

### 3.5. Neurological Outcome and Survival Time after HI Injury

Seven out of 8 perinatal studies reported improved neurological outcomes with creatine treatment. Of these, 3/7 reported improvements in neuropathology without assessing functional outcomes. Two out of 3 studies assessed outcomes after 24 h [18,27] and 1 study assessed outcomes after 1 week [19]. Two out of 7 reported functional improvements through reduced electrographic seizures during the first 20 min after hypoxia [20,21]. One out of seven studies reported partial improvements in functional outcomes (improved swim speed and distance but not grip strength) but no improvement in histopathology 9 weeks after injury [23]. One of 7 studies reported improvements in both function and histological outcomes 11 weeks after injury [24]. One out of 8 perinatal studies reported no significant improvements in cell pathology or functional outcomes with treatment when assessed at 15 weeks after injury [22]. Importantly, only 1 study included sham control groups that enabled assessment of the impact of creatine on healthy brain tissue, in addition to reporting on the severity of HIE [27]. None of the perinatal studies reported adverse outcomes with creatine treatment.

Nine out of 10 individual adult studies reported improved outcomes with creatine treatment. Of these, 1 study assessed outcomes immediately after HI [33], 4 studies assessed outcomes between 1 and 3 days after HI [31,32,36,37] and 4 studies assessed outcomes at 4-7 days after HI [29,30,35]. Five studies reported improvements in histological and functional outcomes [29,30,31,32,37], 1 reported improved histological outcomes but no improvement in function [29], 2 assessed neuropathology alone [35,36] and 1 assessed functional outcomes alone by measuring oxidative metabolism using magnetic resonance spectroscopy (MRS) [33]. One study reported no significant improvement in neuropathology immediately after HI using diffusion weighted MRI [34].

### 3.6. Sex

One of eight perinatal studies used both sexes [27]. Two studies used only males [22,23] and one study used only females [24]. Four out of eight perinatal studies did not report the sex of the subjects [18,19,20,21]. None of the adult studies reported assessing outcomes in both sexes. Five of nine adult studies used male subjects [29,30,31,33,34], one used females [32], and three studies did not report the sex of the subjects [35,36,37].

### 3.7. Temperature Monitoring

Two of 8 perinatal studies reported measuring core temperature during and after HI, both reported improved outcomes. Of these, 1 study maintained core temperature at 37 °C throughout the recovery period (data not shown) [18] and 1 study reported a core temperature range of 32−35 °C, indicating subjects were hypothermic [20]. In the remaining perinatal studies (*n* = 6), 2 studies showed improvements in functional and or histological outcomes and reported monitoring ambient temperature during HI [19,27] and 4 studies did not report temperature monitoring as part of their experimental protocol [21,22,23,24].

Five out of 9 adult studies measured and controlled core temperature during HI but not during recovery, all of them showed improved outcomes with creatine treatment [29,30,31,33,36]. The remaining adult studies (*n* = 4) did not report measurement or control of core temperature as part of their experimental protocol [32,34,35,37].

### 3.8. Risk of Bias Assessment

Quality assessment of the studies was completed using the SYRCLE RoB tool [17] (Table 4). No studies were assessed to have a low risk of bias across all domains. Only 6/17 studies stated that allocation of animals to groups was random [19,21,27,30,31,36], although none gave specific details relating to how the randomization was performed. Selection bias influenced by baseline characteristics was judged as unclear in 24% (*n* = 4) of studies as these studies did not report age at the time of HI and therefore animal baseline prior to intervention/injury could not be inferred, except for one study where age at the time of insult was not reported but baseline characteristics were reported for measures of neurological function [29]. No studies explicitly reported randomly housing animals during the experiment. Two studies reported blinding of assessors to intervention and control groups [19,27]. Four studies (18%) reported that animals were randomly selected for outcome assessment [19,21,27,37] and 3 studies reported blinding of outcome assessors [19,27,37]. Two studies did not report the number of subjects in the study groups, resulting in a high attrition bias [35,37]. In a separate study, the attrition bias was unclear since the number of subjects was either unclear or not reported for all of the outcomes measures [21]. All studies were free from additional bias as outlined by the SYRCLE RoB tool.

## 4. Discussion

This systematic review of preclinical studies investigating the neuroprotective potential of creatine for HIE indicates that creatine is a highly promising neuroprotective agent when administered before or shortly after HI. A comprehensive appraisal of the relevant 17 preclinical studies revealed that creatine conferred the most consistent neuroprotection when administered prior to injury induction in perinatal and adult studies. Moreover, there appears to be an age-dependent creatine loading capacity in the brain that decreases with advancing age. No studies reported adverse side effects from creatine treatment regardless of age, duration, or route of administration. However, we have identified several important knowledge gaps in the current literature that must be addressed in translational preclinical studies before creatine can be tested in pragmatic clinical trials for perinatal encephalopathy. Of particular concern, the majority of preclinical studies did not control for sex, study long-term histological and functional outcomes, provide adequate body temperature control, or study larger animals considered to be more representative of human pregnancy and brain development, and which are therefore good translational models of perinatal HIE [38,39]. The interaction of creatine with therapeutic hypothermia is also yet to be studied in detail. Furthermore, risk of bias assessment using the SYRCLE risk of bias tool showed that none of the studies had a low risk of bias across all the assessment domains.

### 4.1. Bioavailability

A key consideration relating to efficacy of dietary or systemic creatine administration for neuroprotection is whether direct or maternal creatine administration raises brain or cerebrospinal fluid (CSF) creatine concentration to the level required for neuroprotection. Whilst the brain is capable of synthesizing creatine, the passage of extracerebral creatine into the brain is regulated by the SLC6A8 creatine transporter located on the blood brain barrier [40]. Once in the brain the same creatine transporter located on the plasma membrane of neurons and glia modulates creatine entry into the intracellular compartment [41].

The adult brain has a relatively slow uptake of creatine via the blood brain barrier [42], while the rate of creatine uptake in the perinatal brain is less well defined. Within this systematic review, an age-dependent creatine loading capacity was evident. In rodents and rabbits, all studies at ages less than P10 found consistently increased levels of cerebral creatine/phosphocreatine. On the other hand, in studies conducted at postnatal ages greater than P20, there was either no increase or only a very small increase in cerebral creatine levels. In one study, it was reported that intracerebral creatine and phosphocreatine levels were elevated by approximately 50% within 6 h of intraperitoneal creatine injection in P7 rats [19]. In P5-P30 rabbit pups, Holtzman et al. reported that the capacity for endogenous brain PCr/NTP loading was inversely related to age, such that pups aged between P5 and P20 showed greater cerebral creatine loading compared to P30 pups [21]. Moreover, studies conducted in adult rodents found no changes in creatine/phosphocreatine loading in brain tissue following 3-4 weeks of dietary supplementation [30], whereas 10 days of dietary creatine supplementation resulted in a small increase (7–13%) in total brain creatine or creatine/choline (20%) concentration [33,34]. These finding suggests that there may be a greater capacity for creatine transport across the blood brain barrier in the developing brain compared to the mature blood brain barrier. Maturational differences in creatine transporter (SLC6A8) expression within the blood brain barrier have been demonstrated in a series of elegant studies by Braissant et al., where low permeability of creatine across the blood brain barrier was associated with a lack of SLC6A8 expression on the astrocytic feet around microcapillary endothelial cells (neurovascular unit) and choroid plexus [43]. As such, most of the creatine in the adult brain is thought to be derived through intra-cerebral synthesis [43]. By contrast, the fetal/perinatal brain expresses high levels of SLC6A8 throughout the choroid plexus and neurovascular unit [44]. Collectively, these data suggest the age dependent differences in creatine transporter expression throughout the blood brain barrier determine the efficacy of creatine transport between the periphery and central nervous system. Furthermore, the duration of creatine supplementation may determine the degree of cerebral creatine loading into the brain, and therefore its efficacy as a neuroprotectant. Indeed, long term creatine supplementation has been associated with reduced expression of creatine transporter antibodies in skeletal muscle [45]. However, the anti-creatine transporter antibodies used in this study were later shown to be cross-reactive with non-creatine transporters, and hence these data should be interpreted with caution [46]. In P1 spiny mice, mRNA expression of the creatine transporter (SLC6A8) did not differ in the brain tissue from neonates of mothers fed a 5% creatine supplemented diet from mid-gestation (gestational day 20) to term (gestational day 39) [47]. Nevertheless, whether prolonged creatine supplementation and pathological events, experienced during the antenatal and or postnatal period, affect creatine transport across the blood brain barrier and or the distribution and density of creatine transporters on the blood brain barrier, neurons and glia remains to be determined.

Indeed, it is interesting to note that only 8 out of 17 studies assessed creatine levels within the brain. Two of these studies reported neuroprotection without increased brain creatine content [30,32]. In the absence of increased cerebral creatine loading, creatine may promote neuroprotection via its uptake into vascular endothelium and/or smooth muscle cells to improve cerebrovascular function during and after HI [30]. Furthermore, creatine has been shown to have anti-inflammatory and anti-oxidative properties [48,49,50,51]. Thus, modulation of cerebrovascular function, systemic inflammation and or oxidative stress pathways may be other potential mechanisms through which creatine may promote neuroprotection in the absence of increased cerebral creatine loading.

The effectiveness of maternal administration of creatine pre-supposes that it is efficiently transferred across the placenta. For at least one animal used in perinatal research (sheep) this has been shown not to be the case [52]. The instrumented fetal sheep provides a very powerful translational model for understanding the pathophysiology of perinatal brain injury and translating effective therapeutic interventions [53,54]. However, as herbivores, sheep do not ingest creatine to any great extent, it may be that epithelial barriers in such species have lost the ability to transfer creatine across the placenta. Thus, studies of creatine in pregnancy need to either be undertaken in species where creatine has been shown to cross the placenta (e.g., rat [55], spiny mouse [47], human [56]) or account for species differences in creatine transfer by directly administering creatine to the fetus or neonate.

The use of creatine analogues has emerged as a potential means for bypassing limitations in creatine transport across the blood brain barrier conferred by the creatine transporter. Although creatine analogues were excluded from this review, due to potential off target effects, several studies have reported neuroprotection with creatine analogues in rodent models of HI [57,58,59]. However, intravenous boluses of creatine analogues have been associated with neurotoxicity [60], raising potential concerns around the safety of creatine analogues for perinatal neuroprotection. Nevertheless, there is merit in further preclinical study of creatine analogues in relation to HIE to evaluate their safety and to determine if bioavailability and potential neuroprotective effects of creatine can be further optimized.

### 4.2. Regime and Timing of Delivery

One of the key translational considerations for testing potential neuroprotectants is when to treat. The administration regimes for creatine were variable. However, most of the perinatal and adult studies surveyed started the intervention before HI and reported improved pathological and/or functional outcomes. The rationale for pre-treatment is most likely based on the early adult studies that determined a period of pre-treatment was necessary for a protective effect to become manifest [33], and therefore it has been considered necessary in perinatal studies to provide a pre-HI exposure of days to weeks. Only three perinatal studies and one adult study tested creatine after the insult. All three perinatal studies tested dietary creatine supplementation (1–3%) administered from 10 days after inducing HI in P10 mice. The outcomes were mixed, with one study reporting no improvement in histological outcomes but improvements in cognition and sensory-motor function [23], another study reporting improved histological outcomes and sensory motor function [24], and one study reporting no significant improvements in either histological or functional outcomes [22]. The only adult study that tested post-insult treatment administered creatine into the brain via intracerebroventricular infusion 30 min after HI and reported a reduction in hippocampal injury but no improvement in sensory-motor function [29]. The mechanisms underlying cell death, which occur during or very soon after HI, are different from those that occur in the hours, days, and weeks after the insult [61,62]. Therefore, any effects arising from pre-treatment or immediate post-insult treatment do not necessarily indicate the treatment will be effective if the injury had already started to evolve. Clinically, it remains difficult to identify fetuses or neonates who are at risk of developing encephalopathy in advance since the positive predictive value of fetal heart rate monitoring and biophysical profiling for predicting adverse perinatal/neurodevelopmental outcomes is low [63,64]. Therefore, the prophylactic use of creatine has been considered the most important strategy for future clinical trials, which is supported by the collective findings of the studies in this systematic review. Whether prophylactically treating all pregnancies with creatine is a safe and pragmatic option is in the early phase of clinical trials (ACTRN12620001373965). Pharmacological interventions for HIE need to be administered around the time of bulk cell death, which occurs within hours to days after the insult, to maximize their neuroprotective efficacy [54,61,62,65,66]. Collectively, these data indicate that we must further improve our understanding of the therapeutic window that is most likely to demonstrate benefit, in carefully designed preclinical studies, before progressing creatine further down the translational pipeline.

### 4.3. Survival Time

One of the main limitations of the studies identified in this systematic review is that the majority (14/17, 82%) used survival times of ≤7 days. Indeed, 50% of perinatal studies used survival times of ≤1 day. Only three perinatal studies assessed outcomes beyond 1 week; however, none of them reported outcomes in both sexes, and core temperature control was either not reported or not performed after the insult. Neurological outcomes were mixed, such that histological outcomes did not correlate with functional outcomes [23]. Further, the study that used the longest survival time (16 weeks) reported no significant improvements in histological or functional outcomes [22]. Thus, further preclinical studies focusing on the long-term histological and functional impacts of creatine are needed.

### 4.4. Sex

Only one out of eight perinatal studies surveyed reported outcomes from both males and females. Interestingly, this study also reported a significantly higher post HI mortality rate in males compared to females (*p* < 0.01), and a greater benefit of creatine supplementation for male survival [27]. The remaining perinatal and adult studies (*n* = 16 out of 17) either studied one sex or did not report the sex of the subjects. Preclinical and clinical studies have reported sexual dimorphisms in the severity and evolution of perinatal encephalopathy and responses to treatment [67,68,69,70]. There are limited data indicating sex-specific differences in creatine uptake in the adult brain [71]. However, whether these differences exist in the perinatal brain and are physiologically significant enough to impact on HIE should be considered. Overall, these data raise the need for a greater emphasis on evaluating the impact of sex in future preclinical investigations of creatine for neuroprotection.

### 4.5. Creatine for Neuroprotection in the Era of Therapeutic Hypothermia

Most of the perinatal studies (6/8) used neonatal mice or rats at P7-P20. Only one study reported measuring body temperature during and after the insult, albeit the reported temperature range was 32–35 °C, indicating that subjects were hypothermic [20]. The remaining studies measured ambient temperature as a surrogate for body temperature; however, 50% of perinatal studies (*n* = 4/8) only measured ambient temperature during HI. This is an important consideration since normal healthy neonatal rodents show lower body temperatures in typical laboratory nesting conditions between P7 and P14 [72]. For example, the core temperature of neonatal rodents was shown to be affected by the amount and composition of nesting material, position in the nest, huddling, distance from the dam, and the time since the last period of suckling [72].

Four out of nine adult studies did not report measuring temperature during HI. None reported temperature monitoring after the insult. In adult rodents, the use of anaesthetics, such as halothane, isoflurane, and sodium pentobarbital, around the time of HI has been shown to induce hypothermia, as previously reviewed [73]. Furthermore, several rodent studies have reported that the beneficial effects of potential neuroprotectants were confounded by hypothermia during the first 6–8 h after the insult and that maintenance of normothermia abolishes neuroprotection [39,73,74,75]. To the best of our knowledge, the effects of creatine on thermoregulation have not been studied thoroughly and are virtually unknown for neonates. Creatine has been associated with increased peripheral perfusion after tissue ischaemia [76]. Therefore, if body temperature is not carefully controlled, it is reasonable to speculate that creatine may affect thermoregulation via increased radiation to the environment, particularly in neonatal rodents with a small body mass relative to surface area, lack subcutaneous fat, naked skin, poor control of peripheral vasculature, and who do not shiver [77]. In this regard, studies in rodents have shown neuroprotective effects of pharmacological interventions, such as magnesium sulphate, were associated with mild hypothermia and that neuroprotective effects were largely abolished if normothermia was maintained [74].

Collectively, these data demonstrate that effective monitoring and control of core temperature is essential in future preclinical assessments of creatine for neuroprotection to avoid confounding effects of hypothermic conditions and iatrogenic hypothermia. This will allow us to understand the mechanism/s by which creatine per se induces neuroprotection and evaluate its potential for individuals that may not undergo cooling for neuroprotection (e.g., babies born <35 weeks of gestation or individuals born in low-resource settings). Nevertheless, given that therapeutic hypothermia is now standard clinical practice for near-term/term infants with moderate to severe HIE, testing the effectiveness of creatine as an adjuvant to therapeutic hypothermia to improve current treatment protocols is yet to be studied and warrants investigation.

### 4.6. Bias

To examine study bias, we used the SYRCLE risk of bias assessment tool. Most studies (11/17) were unclear in their methods for concealing the allocation sequence of study subjects, 2/17 studies did not report blinding of caregivers and/or examiners from knowing which intervention each subject received, and 14/17 studies did not clearly report the completeness of outcome data, including attrition and exclusions. Furthermore, most studies did not monitor body temperature after HI, raising the possibility that neuroprotection was associated with confounding mild hypothermia rather than creatine treatment alone. Collectively, these data highlight important inconsistencies in the quality of the data surveyed. It is not possible to know if the methodological issues identified in our analysis affected the outcomes of the studies. However, if this critical information is not reported or accounted for in publications, it makes it difficult to assess the significance of past and future studies in a meaningful way.

## 5. Conclusions

Our findings suggest creatine is a highly promising neuroprotective intervention for prevention or treatment of HIE in the perinatal and adult brain. However, we have also revealed several key knowledge gaps that must be addressed in future preclinical studies to ensure the successful translation of creatine for perinatal neuroprotection. For example, there is limited evidence for whether the age of the foetus, neonate, or adult and the duration of creatine infusion affects creatine transport across the blood–brain barrier or whether creatine needs to enter the brain to have neuroprotective effects. Many studies did not examine pragmatic treatment regimens after HI, and these data are essential to further define the window of opportunity during which creatine is effective for neuroprotection. Furthermore, reporting and testing the effect of sex, assessing long-term histological and functional outcomes, ensuring adequate control of body temperature, studying large animal translational models of HI, and following guidelines to mitigate study bias [17,78] are essential if creatine is to be successfully translated for perinatal neuroprotection against HIE.

## Figures and Tables

**Table 1:** Literature search terms.

Database	Search Terms Used
PUBMED	(“hypoxia-ischemia, brain”[MeSH Terms]) OR (“hypoxia, brain”[MeSH Terms]) OR (“brain ischemia”[MeSH Terms]) OR (“stroke”[MeSH Terms]) AND (“creatine”[MeSH Terms]) AND (“cerebral”[All Fields]) OR (“brain”[All Fields]) OR (“brain injury”[All Fields]) AND (“english”[Language]) AND (“preclinical”[All Fields]) OR (“animals”[MeSH Terms:noexp]) NOT “review”[Publication Type]
EMBASE	(“hypoxia ischemia” OR “hypoxia” OR “ischemia” OR “stroke”) AND “creatine”:ti, ab AND (“brain”/exp OR “brain injury” OR “cerebral”) AND [animals]/lim AND [english]/lim
OVID MEDLINE	(Hypoxia/or Brain Ischemia/or Hypoxia-Ischemia, Brain/or Hypoxia, Brain/or Stroke/) and Creatine/and Animals/(limited to English language and review articles excluded)

**Table 2:** Perinatal studies of creatine treatment stratified by age of HI insult.

Reference	HI Model		Intervention Characteristics		Outcome Assessment (Compared to Vehicle Treatment + HI Injury)				
	Age; Species; Sex	HI Method	Dose, Route and Frequency	Timing	Temp (T)	Time of Assessment	Pathology	Function	Measurement of Brain Creatine
[27]	38 d gestation Spiny mice; ♀/♂	Birth asphyxia, 7.5 min in 37–38 °C saline bath	5%; maternal diet daily	Pre-HI: 20–38 days of gestation	Ambient T 37–38 °C during HI, heat pad during recovery, T not reported	1 d post HI	~40–55% ↓ apoptosis in cortical subplate, piriform cortex and thalamus 27% ↓ lipid peroxidation in cerebrum vs. vehicle + HI, *p* < 0.05	-	-
[21]	P5, 10, 15, 20, 30, New Zealand white rabbits; ? sex	4% O_2_ for 8 min	3 g/kg s.c. daily	Pre-H: 3 days	-	During and 20 min post H	-	↓ electrographic seizures at P15 vs. vehicle + HI, *p* < 0.05	Cr loading was age dependent: PCr/NTP in Cr group at P5 ~↑ 65%, at P15 ~↑ 60%, at P20 ~↑ 30%, at P30 *↔* vs. vehicle + HI
[18]	P7, Sprague-Dawley rat; ? sex	Right CAL, then 100 min of hypoxia (8% O_2_)	3 g/kg s.c. daily	Pre-HI: 3 days	Core T: 37 °C during HI and recovery	1 d post HI	24% ↓ in brain oedema vs. vehicle + HI, *p* < 0.05	-	P7: PCr/NTP in Cr ~27% ↑ vs. vehicle + HI
[19]	P7, Wistar rat; ? sex	Left CAL, then 80 min of hypoxia (8% O_2_)	3 g/kg s.c. daily	Pre and post HI: −64, −40, −16 and +3 h	Ambient T: 36 °C during HI	7 d post HI	~24% ↑ cerebral hemisphere volume; ↓ neuronal necrosis in the cortex (~23–48%) and hippocampus (CA1–4, DG; ~28–49%) vs. vehicle + HI, *p* < 0.05	-	P7: 49% ↑ Cr vs. vehicle, 45% ↑ PCr vs. vehicle + HI
[20]	P10 and P20, Long Evans rat; ? sex	4% O_2_ for 8 min	3 g/kg s.c. daily	Pre-HI; 3 days	Core T: 32–35 °C during and post HI	During and 20 min post HI	-	P10: ↓ electrographic and behavioural seizures vs. vehicle + HI, *p* < 0.05 P20: No seizures in control and Cr treated groups	P10: 25% ↑ PCr/NTP vs. vehicle, P20: *↔*PCr/NTP vs. vehicle + HI
[23]	P10, Albino (BALB/C) mice; ♂	Right CAL, then 25 min of hypoxia (8% O_2_)	2% dietary supplement daily	Post HI: P20 for 8 weeks	-	9 weeks post HI	↔ infarct size	↑ muscle strength and co-ordination vs. HI + vehicle, *p* < 0.05, ↔ spatial memory	-
[24]	P10, Albino mice; ♀	Left CAL, then 25 min of hypoxia (8% O_2_)	1 or 3% dietary supplement daily	Post HI: started at P20 for 10 weeks	Heat pad 36 °C during HI	11 weeks post HI	37% ↓ infarct size in 3% diet vs. HI + vehicle, *p* < 0.05	↑ sensory motor function and spatial memory vs. HI + vehicle, *p* < 0.05; 3% diet performed better in all parameters	-
[22]	P10, Albino (BALB/C) mice; ♂	Right CAL, then 25 min of hypoxia (8% O_2_)	2%; dietary supplement daily	Post HI: started at P20 for 15 weeks	Heat pad 36 °C during HI	1 h, 24 h and 16 weeks post injury	↔ infarct size	↔ functional neurological scoring in all tests	-

Abbreviations: Cornu ammonis of the hippocampus (CA); carotid artery ligation (CAL); creatine (Cr); dentate gyrus of the hippocampus (DG); hypoxia-ischaemia (HI); middle cerebral artery occlusion (MCAO); not-reported (-); nucleoside triphosphate (NTP); postnatal (P); phosphocreatine (PCr); subcutaneous (s.c.).

**Table 3:** Adult studies of creatine treatment stratified by age of HI insult.

Reference	HI Model		Intervention Characteristics		Outcome Assessment (Compared to Vehicle Treatment + HI Injury)				
	Age; Species; Sex	HI Method	Dose, Route and Frequency	Timing; Frequency	Temp (T)	Time of Assessment	Pathology	Functional	Measurement of Brain Creatine
[30]	>7 weeks old; 129S6/ ScEv mice; ♂	Transient MCAO for 45 min	0.5%, 1% or 2%; dietary supplement daily	Pre-HI: 3 weeks	Core T: 37°C during HI and recovery (data not shown)	During injury and 4 days post HI	~35–40% ↓ infarct size: 1 and 2% creatine vs. vehicle + HI, *p* < 0.05; ~41% ↓ ADC in 2% creatine vs. vehicle + HI, *p* < 0.05	~63% ↑ brain perfusion during recovery vs. vehicle + HI, *p* < 0.05	*↔* Cr, PCr
[31]	9–10 weeks old; Sprague-Dawley rat; ♂	Transient MCAO for 2 h (described as internal carotid artery)	20 mg/kg BW; IP	Pre-HI; NS	Core T: 37–37.5 °C during HI	24 h post HI	45% ↓ apoptotic cells in ischemic penumbra; ↓ apoptotic gene expression in penumbra vs. vehicle + HI, *p* < 0.05	46% ↑ functional neurological score vs. vehicle + HI, *p* < 0.05	-
[29]	Adult; Sprague-Dawley rat; ♂	12 min BCAO	50 mM, continuous infusion at 0.25 µL/h; ICV daily	Pre-HI: 5 days	Core T: 37–38 °C during HI	7 days post HI	↓ pyknosis in hippocampus (CA1–3 & DG), neocortex and striatum vs. vehicle + HI, *p* < 0.05	~55% ↑ functional neurological score vs. vehicle + HI *p* < 0.05	-
[29]	Adult; Sprague-Dawley rat; ♂	12 min BCAO	50 mM, continuous infusion at 0.25 µL/h; ICV daily	Post-HI: 7 days	Core T: 37–38 °C during HI	7 days post HI	↓ hippocampal pyknosis vs. HI + vehicle, *p* < 0.05	↔ neurological score	-
[32]	Adult; C57BL/6 mouse; ♀	Transient MCAO for 2 h	2%; dietary daily	Pre-HI: 4 weeks	-	30 min and 24 h post HI	56% ↓ in infarct size; ↓ caspase activation in ischemic brain region vs. vehicle + HI, *p* < 0.05	50% ↑ functional neurological score vs. HI + vehicle, *p* < 0.05 ↔ brain perfusion during recovery	*↔* Cr
[33]	Adult; Wistar rat; ♂	12 min BCAO	2.56 g/kg BW; dietary daily	Pre-HI: 10 days	Core T: 37 °C during HI	During injury and 60, 90 min post HI	-	↑ oxidative metabolism vs. vehicle + HI, *p* < 0.05	13% ↑ tCr
[34]	Adult; Wistar rat; ♂	12 min BCAO	2.23 g/kg BW; dietary daily	Pre-HI: 10 days	-	10 min before, during and 18 min post HI	No significant improvement on MRI during injury and reperfusion	-	7% ↑ tCr
[35]	Adult; Sprague-Dawley rat; ? sex	12 min BCAO	50 mM, continuous infusion at 0.25 µL/h; ICV daily	Pre-HI: 5 days Post-HI: 7 days	-	7 days post HI	~70%↓ in neuronal pyknosis and ↓ gliosis in hippocampus (CA1 and 3), neocortex, and caudate nucleus vs. vehicle + HI, *p* < 0.05	-	-
[36]	Adult; Wistar rat; ? sex	10 min BCAO	150 mg/kg BW; IV	Pre-HI: 60 min	Core T: 37–38 °C during HI	48 h post HI	~53%↓ in apoptosis, ~28% ↓ in lipid peroxidation vs. vehicle + HI, *p* < 0.05	-	-
[37]	Adult; Wistar rat; ? sex	Transient MCAO for 2 h	100, 200 and 400 mg/kg BW; IV	Pre-HI: 30 min	-	24 and 72 h post injury	~25–38% ↓ apoptosis with increasing dosage at 72 h, *p* < 0.05 vs. vehicle + HI; ~20–40% ↓ AQP4 protein vs. vehicle + HI at 72 h, *p* < 0.05	~13–65% ↑ neurological score with increasing creatine dosage at 72 h vs. vehicle + HI, *p* < 0.05	-

Abbreviations: apparent diffusion coefficient (ADC); aquaporin 4 (AQP4); bilateral carotid artery occlusion (BCAO); per body weight (BW); Cornu Ammonis of the hippocampus (CA); dentate gyrus of the hippocampus (DG); hypoxia-ischaemia (HI); intracerebroventricular (ICV); intraperitoneal (IP); intravenous (IV); middle cerebral artery occlusion (MCAO); not-reported (-); magnetic resonance spectroscopy (MRS); phosphocreatine (PCr); total creatine (tCr); X-linked inhibitor of apoptosis (XIAP).

**Table 4:** SYRCLE Risk of Bias Assessment for included studies.

Reference	Selection Bias			Performance Bias		Detection Bias		Attrition Bias	Reporting Bias	Free from Other Bias?
	Random Sequence Generation	Groups Similar at Baseline	Allocation Concealment	Animals Random Housing	Blinding of CAREGIVERS and/or Examiners	Random Outcome Assessment	Blinding of Outcome Assessor	Incomplete Outcome Data Addressed	Free from Selective Outcome Reporting	
[18]	Unclear	Yes	Unclear	Unclear	Unclear	Unclear	Unclear	Yes	Yes	Yes
[24]	Unclear	Yes	Unclear	Unclear	Unclear	Unclear	Unclear	Yes	Yes	Yes
[19]	Unclear	Yes	Yes	Unclear	Yes	Yes	Yes	Yes	Yes	Yes
[20]	Unclear	Yes	Unclear	Unclear	Unclear	Unclear	Unclear	Yes	Yes	Yes
[21]	Unclear	Yes	Yes	Unclear	Unclear	Yes	Unclear	Unclear	Yes	Yes
[23]	Unclear	Yes	Unclear	Unclear	Unclear	Unclear	Unclear	Yes	Yes	Yes
[22]	Unclear	Yes	Unclear	Unclear	Unclear	Unclear	Unclear	Yes	Yes	Yes
[27]	Unclear	Yes	Yes	Unclear	Yes	Yes	Yes	Yes	Yes	Yes
[29]	Unclear	Yes	Unclear	Unclear	Unclear	Unclear	Unclear	Yes	Yes	Yes
[37]	Unclear	Unclear	Unclear	Unclear	Unclear	Yes	Yes	No	Yes	Yes
[33]	Unclear	Unclear	Unclear	Unclear	Unclear	Unclear	Unclear	Yes	Yes	Yes
[35]	Unclear	Yes	Unclear	Unclear	Unclear	Unclear	Unclear	No	Yes	Yes
[30]	Unclear	Yes	Yes	Unclear	Unclear	Unclear	Unclear	Yes	Yes	Yes
[36]	Unclear	Yes	Yes	Unclear	Unclear	Unclear	Unclear	Yes	Yes	Yes
[31]	Unclear	Yes	Yes	Unclear	Unclear	Unclear	Unclear	Yes	Yes	Yes
[34]	Unclear	Unclear	Unclear	Unclear	Unclear	Unclear	Unclear	Yes	Yes	Yes
[32]	Unclear	Unclear	Unclear	Unclear	Unclear	Unclear	Unclear	Yes	Yes	Yes

## Data Availability

Data are available from the authors upon reasonable request.

## Supplementary Materials

The following are available online at https://www.mdpi.com/article/10.3390/cells10112902/s1. Table S1: PRISMA Checklist, Table S2: PRISMA Abstract checklist.
